# Supplementary material for: From sequence to enzyme mechanism using multi-label machine learning
Source: BMC Bioinformatics. 2014 May 19;15:150. doi: 10.1186/1471-2105-15-150 (PMC4229970; doi:10.1186/1471-2105-15-150)
Supplement: Additional file 2 — Java code of ml2db. Additional file ml2db_code.tar.gz contains the Java source code to run the multi-label machine learning experiments and save the results to database. The code’s Javadoc is included. [file 1471-2105-15-150-S2.zip › additional file 2/ml2db/ml2db/doc/test/ml2db/mulan/learn/traintest/EvaluationMetricsTest.html]

EvaluationMetricsTest


JavaScript is disabled on your browser.


- Overview
- Package
- Class
- Use
- Tree
- Deprecated
- Index
- Help

- Prev Class
- Next Class

- Frames
- No Frames

- All Classes

- Summary:
- Nested |
- Field |
- Constr |
- Method

- Detail:
- Field |
- Constr |
- Method


test.ml2db.mulan.learn.traintest

## Class EvaluationMetricsTest

- java.lang.Object
- - junit.framework.Assert
  - - junit.framework.TestCase
    - - test.ml2db.mulan.learn.traintest.EvaluationMetricsTest

- All Implemented Interfaces:
  :   junit.framework.Test

  ---

    

  ```
  public class EvaluationMetricsTest
  extends junit.framework.TestCase
  ```

  Tests the evaluation metrics results: accuracy, precision, recall etc.

  Version:
  :   2 May 2011

  Author:
  :   Luna De Ferrari ldf(at)st-andrews.ac.uk

- - ### Constructor Summary

    Constructors

    Constructor and Description

    `EvaluationMetricsTest()`
  - ### Method Summary

    Methods

    | Modifier and Type | Method and Description |
    | --- | --- |
    | `void` | `checkDouble(java.lang.String column, java.lang.Double value)` |
    | `static int[]` | `getFilteredArffs(int trainArffId, int oldArff)` public static void basicPredictionsTest(TrainTestExperimenter exp, String expectedPredictions) { // emit predictions int train = exp.getInputManager().getTrainArffRecord().getArffId(); int test = exp.getInputManager().getTestArffRecord().getArffId(); String machLearningDb = exp.getOutputManager().getExperimentDbManager() .getDatabaseName(); System.out.println("Now predicting from:"); System.out.println("train " + train + ", test " + test + ", results written on db: " + machLearningDb); String predictionsFilePath = PredictionsManager.run(train, test, TestProjectParameters.getDbManagers(), TestProjectParameters.TEST\_PREDICTIONS\_FOLDER, MulanLearners.getBRk1NNAL(), false); // read predictions from file System.out.println("Predictions at: " + predictionsFilePath + "\n\n"); assertEquals(expectedPredictions, FileUtils.getStringFromFile(predictionsFilePath)); } |
    | `static int` | `getInstance1_2_empty_ArffId()` |
    | `static int` | `getInstance1_2ArffId()` |
    | `static int` | `getInstance3ArffId()` |
    | `static void` | `testMetrics_Train_1_2_empty_Test_3(uk.ac.standrews.utils.main.database.TableRow row)` |
    | `static void` | `testMetrics_Train_1_2_Test_3(uk.ac.standrews.utils.main.database.TableRow row)` |
    | `static void` | `testMetricsTrain_1_2_Test_1_2(uk.ac.standrews.utils.main.database.TableRow row)` |

    - ### Methods inherited from class junit.framework.TestCase

      `countTestCases, getName, run, run, runBare, setName, toString`
    - ### Methods inherited from class junit.framework.Assert

      `assertEquals, assertEquals, assertEquals, assertEquals, assertEquals, assertEquals, assertEquals, assertEquals, assertEquals, assertEquals, assertEquals, assertEquals, assertEquals, assertEquals, assertEquals, assertEquals, assertEquals, assertEquals, assertEquals, assertEquals, assertFalse, assertFalse, assertNotNull, assertNotNull, assertNotSame, assertNotSame, assertNull, assertNull, assertSame, assertSame, assertTrue, assertTrue, fail, fail, failNotEquals, failNotSame, failSame, format`
    - ### Methods inherited from class java.lang.Object

      `equals, getClass, hashCode, notify, notifyAll, wait, wait, wait`

- - ### Constructor Detail


    - #### EvaluationMetricsTest

      ```
      public EvaluationMetricsTest()
      ```
  - ### Method Detail


    - #### checkDouble

      ```
      public void checkDouble(java.lang.String column,
                     java.lang.Double value)
      ```


    - #### getFilteredArffs

      ```
      public static int[] getFilteredArffs(int trainArffId,
                           int oldArff)
      ```

      public static void basicPredictionsTest(TrainTestExperimenter exp, String
      expectedPredictions) {
      // emit predictions int train =
      exp.getInputManager().getTrainArffRecord().getArffId(); int test =
      exp.getInputManager().getTestArffRecord().getArffId(); String
      machLearningDb = exp.getOutputManager().getExperimentDbManager()
      .getDatabaseName();
      System.out.println("Now predicting from:"); System.out.println("train " +
      train + ", test " + test + ", results written on db: " + machLearningDb);
      String predictionsFilePath = PredictionsManager.run(train, test,
      TestProjectParameters.getDbManagers(),
      TestProjectParameters.TEST\_PREDICTIONS\_FOLDER,
      MulanLearners.getBRk1NNAL(), false);
      // read predictions from file System.out.println("Predictions at: " +
      predictionsFilePath + "\n\n");
      assertEquals(expectedPredictions,
      FileUtils.getStringFromFile(predictionsFilePath)); }


    - #### getInstance1\_2\_empty\_ArffId

      ```
      public static int getInstance1_2_empty_ArffId()
      ```


    - #### getInstance1\_2ArffId

      ```
      public static int getInstance1_2ArffId()
      ```


    - #### getInstance3ArffId

      ```
      public static int getInstance3ArffId()
      ```


    - #### testMetrics\_Train\_1\_2\_empty\_Test\_3

      ```
      public static void testMetrics_Train_1_2_empty_Test_3(uk.ac.standrews.utils.main.database.TableRow row)
      ```


    - #### testMetrics\_Train\_1\_2\_Test\_3

      ```
      public static void testMetrics_Train_1_2_Test_3(uk.ac.standrews.utils.main.database.TableRow row)
      ```


    - #### testMetricsTrain\_1\_2\_Test\_1\_2

      ```
      public static void testMetricsTrain_1_2_Test_1_2(uk.ac.standrews.utils.main.database.TableRow row)
      ```


- Overview
- Package
- Class
- Use
- Tree
- Deprecated
- Index
- Help

- Prev Class
- Next Class

- Frames
- No Frames

- All Classes

- Summary:
- Nested |
- Field |
- Constr |
- Method

- Detail:
- Field |
- Constr |
- Method
